# Peer support and improved quality of life among persons living with HIV on antiretroviral treatment: A randomised controlled trial from north-eastern Vietnam

**DOI:** 10.1186/1477-7525-10-53

**Published:** 2012-05-18

**Authors:** Vu Van Tam, Mattias Larsson, Anastasia Pharris, Björn Diedrichs, Hoa Phuong Nguyen, Chuc Thi Kim Nguyen, Phuc Dang Ho, Gaetano Marrone, Anna Thorson

**Affiliations:** 1 Division of Global Health, Department of Public Health Sciences, Karolinska Institute, Stockholm, Sweden; 2 Health System Research Project, Ha Noi Medical University, Ha Noi, Vietnam; 3 Department of Infectious diseases, Uong Bi general hospital, Uong Bi, Quang Ninh, Vietnam; 4 Department of Probability and Mathematical Statistics, Institute of Mathematics, Ha Noi, Vietnam; 5 Oxford University Clinical Research Unit, Ha Noi, Vietnam; 6 Sundsvall-Härnösand regional hospital, Sundsvall, Sweden

**Keywords:** Quality of life, Peer support, HIV, ART, Quang Ninh, Vietnam, Randomised controlled trial

## Abstract

**Background:**

Among people living with HIV (PLHIV) on antiretroviral therapy (ART), it is important to determine how quality of life (QOL) may be improved and HIV-related stigma can be lessened over time. This study assessed the effect of peer support on QOL and internal stigma during the first year after initiating ART among a cohort of PLHIV in north-eastern Vietnam.

**Methods:**

A sub-sample study of a randomised controlled trial was implemented between October 2008 and November 2010 in Quang Ninh, Vietnam. In the intervention group, participants (n = 119) received adherence support from trained peer supporters who visited participants’ houses biweekly during the first two months, thereafter weekly. In the control group, participants (n = 109) were treated according to standard guidelines, including adherence counselling, monthly health check and drug refills. Basic demographics were measured at baseline. QOL and internal stigma were measured using a Vietnamese version of the WHOQOL-HIVBREF and Internal AIDS-related Stigma Scale instruments at baseline and 12 months. T-tests were used to detect the differences between mean values, multilevel linear regressions to determine factors influencing QOL.

**Results:**

Overall, QOL improved significantly in the intervention group compared to the control group. Among participants initiating ART at clinical stages 3 and 4, education at high school level or above and having experiences of a family member dying from HIV were also associated with higher reported QOL. Among participants at clinical stage 1 and 2, there was no significant effect of peer support, whereas having children was associated with an increased QOL. Viral hepatitis was associated with a decreased QOL in both groups. Lower perceived stigma correlated significantly but weakly with improved QOL, however, there was no significant relation to peer support.

**Conclusion:**

The peer support intervention improved QOL after 12 months among ART patients presenting at clinical stages 3 and 4 at baseline, but it had no impact on QOL among ART patients enrolled at clinical stages 1 and 2. The intervention did not have an effect on Internal AIDS-related stigma. To improve QOL for PLHIV on ART, measures to support adherence should be contextualized in accordance with individual clinical and social needs.

## Background

The HIV epidemic in Vietnam is in a concentrated stage, with an estimated HIV prevalence of 0.53% in 2010 [1]. Antiretroviral therapy (ART) has been scaled-up in Vietnam since late 2005 with funding through programs such as the US President’s Emergency Plan for AIDS Relief (PEPFAR) and Global Fund to fight AIDS, Tuberculosis and Malaria (GFATM). By December 2010, about 49,492 persons living with HIV (PLHIV) in Vietnam had access to free ART [2].

The world-wide scale-up of ART has decreased the incidence of new HIV infection and reduced AIDS-related deaths substantially [3]. With an increased prevalence of PLHIV on life-long ART, it is becoming increasingly important to determine which factors contribute to a better quality of life (QOL). While people are living longer, they may be living with increased health-challenges related to HIV disease, the side effects of treatment or emerging concurrent morbidities related to HIV or aging. Hence, despite living longer, individuals may not always be ‘living well’. Quality of life has become an essential outcome to consider in the overall health and well-being of people living with HIV. Whereas it is well documented that ART improves not only clinical outcomes but also QOL within the first year [4,5], conclusions on what other factors (besides the ART itself) can contribute to a higher QOL are diverse [6,7]. Several factors have been identified as contributing to better QOL among PLHIV, including social support [8,9], spiritual well-being [9], education level [10,11], not being an injecting drug user [12,13] and having good adherence to ART [14,15]. Meanwhile, other factors such as HIV-related stigma [16-18], non-disclosure of one’s HIV status have been reported to negatively affect QOL [19]. Due to the strong relationship between QOL and many important indicators for treatment success, QOL has been widely applied in evaluating the impact of HIV-related interventions among different populations [20,21].

In settings with heavy HIV-related stigma and discrimination and limited health care resources, such as Vietnam [22], scaling up HIV care faces challenges, including shortages of health care personnel willing to work with HIV-infected individuals resulting in heavy workloads and constrained support to patient adherence. To counter this, community-based peer support interventions have sought to improve adherence to ART, to lessen internal HIV-related stigma as well as to improve treatment outcomes such as QOL [23]. However, to date, there is no available data evaluating such an intervention in Vietnam.

In this randomized controlled trial, we assess the impact of peer support on QOL and internal stigma for PLHIV after 12 months on ART.

## Methods

### General study design

This study focusing on QOL is a sub-sample of a cluster randomized controlled trial aiming to assess the impact of peer support on viral suppression and resistance development among patients in Quang Ninh province in north-eastern Vietnam (DOTARV).

### Study setting

The study was conducted at four outpatient clinics (OPC) in Quang Ninh, a province in the northeast of Vietnam with a population of 1.1 million. Quang Ninh’s economy is rapidly growing and mainly based on industries such as coalmines, cement plants and harbours as well as tourism within the famous Ha Long Bay. It is also the area that is hardest hit by the HIV epidemic in Vietnam, with an estimated HIV prevalence of slightly above 1% among 15-49-year-olds [24].

### Sampling and participants

The study sample was selected from four districts in Quang Ninh province, which consisted of 71 communes (28 urban and 43 rural). The total population of the 71 communes was 612,541 in 2009. Cluster-based sampling at the level of the commune was employed in order to minimize contamination between patients living near each other. In cluster sampling, the 71 communes were randomised to either intervention (36 communes) or control (35 communes), after an initial matching according to rural–urban, population and vicinity to hospital. In both intervention and control communes, all patients who came from the same commune were then treated similarly in a standardized way according to the study protocol. The study enrolled HIV positive patients who were ARV-naïve and eligible to initiate ART according to the Vietnamese national guidelines at the time of the study. Inclusion criteria were as following: clinical stage 4 of HIV disease (AIDS related illnesses) regardless of CD4+ count, clinical stage 3 (severe opportunistic infections) with CD4+ <350/μl, clinical stage 1 and 2 (asymptomatic or mild infection) with CD4+ count of <200/μl [25] . Exclusion criteria were pregnancy, age under 18 or above 60, mental illness and institutionalization. While the larger DOTARV study began in 2007, the present sub-study focusing on QOL and internal stigma included all DOTARV participants recruited from October 2008 to November 2009. Two-hundred seventy-five participants were consecutively selected from both the intervention and the control groups (i.e. all persons eligible for ART and meeting inclusion criteria from both intervention and control communes were enrolled in this sub-study). Among these, 24 died within six months of ART initiation, twelve patients did not come for the interview at twelve months and eleven dropped out of the study as per compulsory detoxification or arrested due to heroin trafficking. A total of 228 participants responded to the interview both at baseline and at twelve months.

### Control

Those individuals who were randomized to the control arm of the study received standard care as per normal government health care standards for patients initiating ART. This included adherence counselling and readiness training provided by the medical staff of these OPCs at individual level (three times) and at group level (three times) prior to starting ART. Health checks, adherence assessment and drug refills were carried out monthly at the outpatient clinic. All patients would report their obstacles/barriers to ART adherence (if any) to health staff at the OPCs at monthly visits. In case non-adherence to ART was identified by health staff, adherence counselling would be provided instantly on location.

The adherence assessment in this study was based on the self-reports from the patients as well as pills counts. These were both compared to the amount of the pills that should have been taken using the criteria recommended by WHO [26]. The adherence assessment then referred to: good adherence (patient forgot to take doses less than four times per month); moderate adherence (patient forgot to take doses between 4–8 times per month) and poor adherence (patient forgot to take doses more than 8 times per month).

### Intervention

Individuals in the intervention arm of the study received standard care as described above and also received peer support from trained PLHIV who were taking ART. These peers functioned as “external supporters” for patients initiating ART and performed biweekly visits during the initial two months of ART, when drug-taking habits were being formed. After two months, the visits were reduced to once per week (if treatment adherence was good) or intensified to become more frequent (if adherence was poor). To facilitate the peer support activities and ensure that the adherence support was carried out properly, a standardized checklist was developed by the research group together with a group of PLHIV who were on ART. The checklist was used to guide the peer supporter to ask questions in a standardized order and manner. During each visit, the external supporter went through this standardized checklist including questions about general well-being, signs/symptoms since the last visit, psychological problems or adverse drugs reactions as well as adherence to therapy since the last visit. The checklist was only applied in the intervention group and hence it was not used for data collection or for monitoring the effects of the intervention. Patients and family members were encouraged to report all constraints/obstacles to ART adherence. Barriers to ART adherence identified during the visiting were discussed between the peer supporter, the patient and family members to determine a feasible solution and (if necessary) health staff at the outpatient clinic were contacted for advice. Problems identified by peer supporters such as common barriers, suggestions for changing dose-taking schedules, behaviour of family member towards peer supporter (if any) were discussed among the research group at monthly meetings.

### Measurement tools

Study tools administered to both intervention and control participants included:

**The WHOQOL-HIVBREF** includes questions respond to the definition of Quality of Life as *Individuals’ perception of their position in life in the context of the culture and value system in which they live and in relation to their goals, expectation, standards and concerns.* This measurement produced scores on the patients’ self-reported judgement of six different domains of QOL including: Physical (4 facets: pain, energy, sleep, symptoms); Psychological (5 facets: positive feelings, cognitive, self- esteem, bodily image, negative feelings); Social Relationships (4 facets: personal relationships, social support, sexual activity, social inclusion); Level of Independence (4 facets: mobility, daily activities, dependence on medication, work capacity); Environment (8 facets: physical safety and security, home environment, financial resources, health and social care, opportunities for acquiring new information, opportunity for leisure activities, physical environment, transport) and Spirituality/Personal Beliefs (4 facets: forgiveness and blame, concern about the future, death, spirituality). The patients answered each question using a 5-level Likert scale. Among these, scores of questions with negative direction (negative feelings, pain and discomfort, dependence on medicine, death) were reversed to make higher scores generally indicate better QOL. The score of each domain ranged between 4 and 20. These scores could also be added up to produce an overall score. The higher scores indicated better QOL [27]. The difference between the score at 12 months and the score at baseline was then used to express the change in QOL (both for each separate domain and for the overall score). We strictly followed the protocol provided by the WHOQOL-HIV Group to translate and using forward-backward translation with subsequent reviews and discussions within the research groups.

**The Internal AIDS-Related stigma** questionnaire focused on self-blame and concealment of HIV status [28]. This measurement assessed if patients agreed with statements including: It is difficult to tell people about my HIV infection; Being HIV positive makes me feeling dirty; I feel guilty that I am HIV positive; I am ashamed that I am HIV positive; I sometimes feel worthless because I am HIV positive; and I hide my HIV status from others. Participants responded to each question by agree = 1 or disagree = 0. The total scores ranged from 0 to 6. Lower scores at 12 months means lessened stigma over time.

Both WHOQOL-HIVBREF and Internal AIDS-related stigma measurement tools were pre-tested, revised and validated prior to beginning data collection.

**Baseline characteristics** of the participants were collected through a baseline questionnaire that included socioeconomic characteristics (age, sex, education level, occupation, marital status, number of children, housing, income) and HIV-related characteristics (HIV transmission routes, the duration of knowing their HIV status, other family member infected with HIV or died from HIV, alcohol and drug use behaviours, hepatitis co-infection, clinical staging).

Data on QOL and internal AIDS-related stigma were collected in a separate room at the outpatient clinic through self-administered questionnaires after participants were provided with instructions on how to fill them in by a member of the health staff. These assessments were carried out at initiation of treatment and then every four months in connection to the participants’ scheduled monthly drug pick-up with a planned follow-up time of one year.

### Statistical analysis

Data collected were processed and analysed using SPSS version 13 and STATA version 10. Proportions, means and standard deviations (SDs) were used for the descriptive analysis. Chi-square tests were performed to examine the difference between proportions (sex, age distribution, marital status, occupations, education level, current and past IDU, clinical stage, member of family infected with HIV or died from HIV). WHOQOL-HIVBREF scores and stigma scale scores were assessed for normal distributions. T-tests were used to detect the difference between mean values of QOL scores or Stigma scores in both related samples model and independent samples model. Pearson’s correlation coefficient was used to evaluate the correlation between quantitative variables [29]. Stepwise multiple linear regression and multilevel linear regression methods were used to estimate the causal relationship between QOL change between baseline and 12 months and independent variables. We used default cut-offs provided by SPSS in stepwise multiple linear regression (0.05 to enter in the model and 0.10 to be removed from the model) to choose the most influential independent variables. Then multilevel linear regression was applied to justify the effects of intra cluster correlation. Intra cluster correlation coefficients (ICCs) were calculated to evaluate the similarity of QOL within clusters (communes). In all the tests and regression models, p-values less than 5% were considered significant. Longitudinal approach was attempted in order to take the values of the QOL at different time points (baseline, 4 months, 8 months, 12 months) into account. However, due to the small sample size, the change in QOL during the 4 months intervals was not significant. Thus, only the results related to QOL at baseline and 12 months are presented here.

## Results

Among 228 ARV-naïve patients recruited to the QOL study within the DOTARV project from October 2008 to November 2009, there were 119 in the intervention group and 109 in the control group. Characteristics of the study participants at baseline are described in Table 1. There were no significant differences in these characteristics between the two groups.

**Table 1:** Baseline characteristics of participants enrolled.

**Characteristics**	**Intervention (n = 119)** **%**	**Control (n = 109)** **%**	p-value*
**Age (year)**			
≤ 35	65.5	73.4	0.119
> 35	34.5	26.6	
**Sex**			
Male	65.5	70.6	0.410
Female	34.5	29.4	
**Education**			
Secondary or less	45.4	52.3	0.297
High school or higher	54.6	47.7	
**Occupations**			
Unemployed	16	23.9	0.135
Employed	84	76.1	
**Marital status**			
Widow	12.6	15.6	0.490
Single	31.1	27.5	
Divorced/separated	13.4	8.3	
Married	42.9	48.6	
**Income/month** (USD)			
≤ 30 USD	22	32.1	0.072
> 30 USD	78	67.9	
**Having children**			
Yes	48.7	54.1	0.416
No	51.3	45.9	
**Other PLHIV in family**			
Yes	39.5	38.5	0.882
No	60.5	61.5	
**Someone in family died of AIDS**			
Yes	26.9	20.2	0.234
No	73.1	79.8	
**Reported mode of HIV infection**			
Having sex	52.1	52.8	0.320
IDU	36.1	42.2	
Do not know	11.8	5.0	
**History of IDU**			
Yes	47.1	45.9	0.858
No	52.9	54.1	
**Current IDU**			
Yes	9.2	5.5	0.283
No	90.8	94.5	
**Hepatitis C and/or B co-infection**			
Yes	28.6	22.9	0.332
No	71.4	71.1	
**Clinical staging**			
Stage 1 or 2	47.1	48.6	0.813
Stage 3 or 4	52.9	51.4	

*: Chi-square test in proportions comparison.

### QOL in the intervention and control groups

Overall, QOL of the whole cohort seemed to increase over time, with a mean score of 76.5 at baseline and 77.3 after one year of ART, but this difference was not significant (p = 0.295). However, stratification by intervention–control groups and clinical stages showed different patterns.

Table 2 shows the results of the QOL scores that changed over time within each group. In the intervention group, overall QOL scores and QOL scores of physical and independent capacity increased mainly among patients who presented at clinical stages 3 and 4. Among patients who presented at clinical stages 1 and 2, QOL scores increased slightly in independent capacity (p = 0.033) but decreased in the domain of environment (p = 0.001). In the control group, QOL increased only in independent capacity among patients presented at clinical stages 3 and 4.

**Table 2:** Change in QOL score after 12 months of ART, by QOL domains and intervention versus control group

**Clinical stage**	**QOL by domain**	**Control group**			**Intervention group**		
		**At baseline Mean (SD)**	**After 12 months Mean (SD)**	P-value*	**At baseline ** **Mean (SD)**	**After** **12 months Mean (SD)**	P-value*
Clinical 1 & 2	Physical	12.87 (2.82)	13.57 (1.65)	0.087	13.45 (2.43)	13.87 (2.04)	0.274
	Psychological	12.53 (2.71)	13.05 (1.69)	0.123	13.19 (2.13)	12.93 (1.9)	0.425
	Level of Independence	11.93 (2.34)	12.67 (1.72)	0.061	12.46 (1.68)	13.18 (1.93)	**0.033**
	Social Relationships	12.72 (2.44)	12,31 (1.63)	0.166	12.79 (2.18)	12.36 (1.68)	0.206
	Environment	12.44 (2.56)	12.24 (2.04)	0.590	13.29 (2.31)	12.19 (1.85)	**0.001**
	Spirituality/Personal Beliefs	13.11 (3.34)	13.89 (2.89)	0.272	13.18 (3.61)	13 (3.06)	0.654
	**Overall QOL Scores**	**75.61 (12.65)**	**77.74 (7.77)**	0.337	**78.35 (10.62)**	**77.53 (9.05)**	0.533
Clinical 3 & 4	Physical	12.76 (2.21)	13.04 (2.08)	0.419	12.51 (2.56)	14.16 (1.90)	**< 0.001**
	Psychological	13.0 (1.83)	12.26 (2.11)	0.051	12.69 (2.52)	12.70 (1.89)	0.970
	Level of Independence	11.71 (1.76)	12.47 (2.10)	**0.010**	11.52 (2.05)	13.29 (2.09)	**< 0.001**
	Social Relationships	12.44 (1.92)	12.1 (1.76)	0.491	12.98 (2.45)	12.37 (1.29)	0.073
	Environment	12.44 (1.93)	11.91 (1.79)	0.107	12.66 (2.31)	12.4 (2.12)	0.412
	Spirituality/Personal Beliefs	14.47 (3.17)	13.36 (2.92)	0.107	13.03 (2.85)	13.78 (2.47)	0.14
	**Overall QOL Scores**	**76.82 (8.26)**	**75.36 (9.6)**	0.438	**75.39 (10.38)**	**78.69 (8.47)**	**0.023**

*: T-test for mean comparison of related samples.

Overall QOL score ranged from 24–120.

Table 3 shows the results of comparison of QOL scores changed over time between groups. Among participants enrolled with more severe immunosuppression at baseline (clinical stage 3 and 4), there was a significant association between peer support and improved overall QOL (p = 0.034), more specifically the QOL domains of physical well-being (p = 0.007), level of independence (p = 0.038) and spirituality (p = 0.029). Meanwhile, among participants those were less symptomatic when beginning ART (clinical stage 1 or 2), there were no significant differences between the two groups in overall QOL or in any of the specific domains (Table 3).

**Table 3:** Mean of difference after 12 months of ART between groups

	***Clinical 1 & 2***			***Clinical 3 &4***		
	**Control** **Mean (SD)**	**Intervention** **Mean (SD)**	P-value*	**Control** **Mean (SD)**	**Intervention** **Mean (SD)**	P-value*
**QOL**						
Physical	0.6792 (2.83)	0.4286 (2.90)	0.649	0.3036 (2.78)	1.6508 (2.58)	**0.007**
Psychological	0.5585 (2.59)	−0.2571 (2.39)	0.090	−0.7571 (2.84)	0.0127 (2.66)	0.130
Level of Independence	0.6792 (2.57)	0.7143 (2.44)	0.942	0.8214 (2.28)	1.7619 (2.55)	**0.038**
Social Relationships	−0.5283 (2.73)	−0.4286 (2.50)	0.843	−0.2321 (2.5)	−0.6190 (2.69)	0.421
Environment	−0.2170 (2.91)	−1.0982 (2.36)	0.085	−0.5089 (2.32)	−0.2540 (2.44)	0.562
Spirituality/Personal Beliefs	0.5660 (3.70)	−0.1786 (2.96)	0.248	−0.8715 (4.00)	0.7460 (3.96)	**0.029**
**Overall QOL**	**1.7377 (13.06)**	**−0.8196 (9.76)**	0.248	**−1.2482 (11.95)**	**3.2984 (11.21)**	**0.034**

*: T-test for mean comparison of independent samples.

QOL: higher score: better QOL.

### Factors influencing QOL improvement

Table 4 presents factors related to overall QOL by Univariate analysis. All the factors included in Table 4 which had a p-value < 0.20 (showing a possible correlation with the main outcome) were added in regression models. After stepwise multiple linear regressions, the most influencing independent variables were taken into of multilevel linear regression analysis with individuals as units of level 1 and communes as units of level 2 (Table 5). Intra cluster correlation coefficients (ICCs) of 12-months changing of overall QOL as well as of all domains are presented in Table 5.

**Table 4:** Changing of **Overall QOL Scores** after 12 months of ART and influencing factors by Univariate analysis

**Factors**		**Patients at Clinical stage 1 & 2**		**Patients at Clinical stage 3 & 4**	
		**Mean of difference**	P value*	**Mean of difference**	P value*
**Sex**	Male	−1.1149	0.078	0.2227	0.114
	Female	2.8786		3.8161	
**Age**	>35	−1.3867	0.314	2.1975	0.495
	= < 35	1.1114		0.6329	
**Occupation**	Unemployed	3.3500	0.24	1.4333	0.891
	Employed	−0.1549		1.1775	
**Education**	Secondary or less	1.7457	0.3080	−1.0789	**0.046**
	High school or higher	−0.5413		3.2161	
**Having children**	Yes	2.5875	**0.033**	1.2738	0.913
	No	−2.1327		1.0379	
**Income/month**	≥30USD	3.2469	0.086	1.2500	0.958
	<30USD	−0.9197		1.1220	
**Other PLHIV in the family**	Yes	1.2143	0.520	3.1725	0.184
	No	−0.2217		0.1392	
**Someone in family died of AIDS**	Yes	0.7192	0.882	7.4926	**0.001**
	No	0.3313		0.7000	
**Own a house**	Yes	3.6938	0.069	3.2419	0.253
	No	−0.6671		0.4126	
**Social support**	High	0.3203	0.919	2.3629	0.245
	Low	0.5464		−0.1509	
**Hepatitis C and/or B**	Yes	−5.9522	**0.002**	−2.3833	**0.034**
	No	2.3880		2.6185	
**Alcohol use**	Yes	−1.0579	0.295	0.7488	0.786
	No	1.5386		1.3727	
**History IDU**	Yes	−0.6667	0.356	0.0873	0.358
	No	1.3828		2.0797	
**Current IDU**	Yes	1.8875	0.711	−1.400	0.499
	No	0.3079		1.3682	
**Intervention**	Yes	−0.8196	0.248	3.2984	0.034
	No	1.7377		−1.2482	

*: T-test for mean comparison of independent samples.

**Table 5:** QOL change after 12 months of ART in multilevel linear regression models

Dependent variable**	**Factors**	**Clinical stage 1 & 2**				**Clinical stage 3 & 4**			
		**ICC (%)**	**Unstandardized Coefficients**	**Standardized Coefficients**	P value*	**ICC (%)**	**Unstandardized Coefficients**	**Standardized Coefficients**	P value*
***Overall QOL***		28.6				42.7			
	Constant		0.979		0.672		−4.690		0.074
	High school or higher education		−2.537	−0.109	0.254		5.311	0.226928	**0.010**
	Someone in family died of AIDS		1.762	0.066	0.487		8.776	0.314378	**0.000**
	Having children		4.561	0.199	**0.036**		−0.486	−0.02076	0.810
	Hepatitis C and/or B co-infection		−6.460	−0.230	**0.017**		−2.764	−0.10861	0.214
	Intervention		−1.001	−0.044	0.689		4.156	0.177439	0.080
***Physical***		32.2				44.7			
	Constant		0.557		0.376		0.420		0.497
	High school or higher education		−0.767	−0.133	0.181		0.556	0.101	0.258
	Someone in family died of AIDS		−0.175	−0.026	0.789		1.204	0.184	**0.037**
	Having children		0.840	0.148	0.132		−0.497	−0.091	0.304
	Hepatitis C and/or B co-infection		−0.204	−0.029	0.773		−1.229	−0.206	**0.021**
	Intervention		0.223	0.039	0.758		1.359	0.247	**0.014**
***Psychological***		27.6				40.9			
	Constant		0.384		0.415		−1.221		**0.037**
	High school or higher education		−0.392	−0.077	0.420		1.013	0.184	**0.044**
	Someone in family died of AIDS		0.722	0.123	0.195		1.411	0.214	**0.018**
	Having children		0.800	0.160	0.091		−0.354	−0.064	0.478
	Hepatitis C and/or B co-infection		−1.199	−0.196	**0.043**		−0.540	−0.090	0.322
	Intervention		−0.495	−0.099	0.414		0.641	0.116	0.232
***Level of independence***		26.1				37.7			
	Constant		0.969		0.056		1.420		**0.010**
	High school or higher education		−0.798	−0.158	0.103		−0.286	−0.058	0.532
	Someone in family died of AIDS		0.509	0.087	0.363		0.431	0.073	0.429
	Having children		0.433	0.087	0.364		−0.551	−0.112	0.226
	Hepatitis C and/or B co-infection		−1.470	−0.241	**0.014**		−0.789	−0.147	0.113
	Intervention		0.347	0.070	0.564		0.806	0.164	0.097
***Social relation***		21.5				33.5			
	Constant		−0.516		0.377		−1.012		0.070
	High school or higher education		−0.304	−0.058	0.561		0.733	0.141	0.125
	Someone in family died of AIDS		−0.148	−0.024	0.840		1.297	0.210	**0.023**
	Having children		0.313	0.060	0.539		0.354	0.069	0.456
	Hepatitis C and/or B co-infection		−1.622	−0.255	**0.011**		−0.261	−0.046	0.615
	Intervention		0.418	0.080	0.472		−0.414	−0.080	0.417
***Environment***		31.2				32.4			
	Constant		−0.182		0.737		−1.155		**0.040**
	High school or higher education		−0.219	−0.041	0.657		0.849	0.179	0.065
	Someone in family died of AIDS		0.213	0.034	0.705		1.096	0.194	**0.038**
	Having children		0.442	0.083	0.358		0.114	0.024	0.799
	Hepatitis C and/or B co-infection		−2.315	−0.355	**0.000**		−0.276	−0.053	0.576
	Intervention		−0.315	−0.059	0.602		0.174	0.037	0.761
***Spirituality/personal beliefs***		22.3				32.0			
	Constant		−0.530		0.444		−3.215		**0.000**
	High school or higher education		−0.051	−0.008	0.939		2.215	0.275	**0.001**
	Someone in family died of AIDS		0.324	0.041	0.671		3.337	0.347	**0.000**
	Having children		1.865	0.279	**0.004**		0.524	0.065	0.446
	Hepatitis C and/or B co-infection		0.348	0.043	0.670		0.522	0.059	0.487
	Intervention		−0.778	−0.116	0.327		1.705	0.211	**0.026**

*: Test to compare regression coefficient to 0.

**: Difference between 12-month score and baseline score.

ICC: Intra cluster (commune) correlation.

#### Results among patients presented at clinical stages 3 and 4

Participants had significant improvement in overall QOL after 12 months if they had higher education (p = 0.01), previously had an experience of a family member dying from HIV (p < 0.001) or received peer support (“borderline” p = 0.080). The influences of intervention and other factors to specific domains are also described in Table 5. People with higher education had significant improvement in Psychological wellbeing (p = 0.044) and Spirituality/personal beliefs (p = 0.001). Meanwhile, experience of a family member dying from HIV gave positive contributions for almost all domains of QOL Conversely, Hepatitis C and/or B co-infection was significantly associated with decreased Physical wellbeing (p = 0.021).

#### Results among patients presenting at clinical stages 1 and 2

Peer support did not appear to have any impact (Table 5). In this group, for those with hepatitis B and/or C co-infection, overall QOL decreased significantly (p = 0.017) after 12 months of follow-up, specifically for the QOL domains of psychological well-being (p = 0.043), level of independence (p = 0.014), social relations (p = 0.011) and environment aspect (p < 0.001). Whilst, having children in family can help to have better Spirituality/personal beliefs (p = 0.004) and then to improve QOL (p = 0.036).

### QOL and internal AIDS-related stigma

The average internal AIDS-related stigma scores for both intervention and control groups) at baseline and after 12 months were 3.21 (SD = 1.96) and 3.27 (SD = 1.80) respectively. The internal AIDS-related stigma did not differ between the intervention and control groups or between the different clinical stage groups after 12 months. There was a significant association between value of QOL change over time and changes in internal AIDS-related stigma (p < 0.001). However, this is not a strong correlation with a correlation coefficient of −0.36. Patients who reported improved QOL after 12 months on ART also reported decreased stigma and vice versa.

## Discussion

This is the first study, to our knowledge, that shows a positive effect of peer support on QOL among severely immunosuppressed patients initiating ART in the context of a randomised controlled trial. We found that peer support had a very different effect on QOL depending on the patient’s clinical condition when starting ART. Those with severe immunosuppression and opportunistic infections (clinical stages 3 or 4) who received extra adherence support from a trained peer supporter reported significantly improved QOL after 12 months on ART compared to a control group who received standard care. This improvement in the intervention group was not found among patients who were asymptomatic or who had mild symptoms (clinical stage 1 or 2) when ART was initiated.

QOL was particularly improved among severely immunosuppressed intervention-group patients in the domains that relate to the clinical condition such as physical well-being, level of independence and spirituality (perceptions about the future or worrying about death). For other QOL domains (psychology, social relationships and environment) improvement appeared to depend on individual factors such as level of education and earlier experience of a family member dying from HIV rather than on contact with a peer supporter. The improvement in QOL in some segments of the intervention group might have been because the peer supporters were able to utilise their own experiences as PLHIV to empathically listen, understand, advise and assist the patients to problem-solve. In addition, as the peer supporters had received training, they could act as intermediaries between patients and health care providers [30], giving information, counselling and assisting patients to contact health staff when needed [23], particularly in cases the patients experienced severe symptoms that could influence QOL negatively [31].

While QOL became better over time among patients started ART in advanced stage of HIV/AIDS (clinical stage 3 or 4), the patients who had not experienced AIDS and opportunistic infections (clinical stage 1 or 2) often showed a decline QOL after baseline [32]. They might have perceived the regular visits of the external supporters as annoying or threatening due to the risk of involuntary disclosure to neighbours, which might have reduced some aspects of QOL [19]. Other explanations such as challenges with starting ART per se, including the treatment associated stigma we found in an earlier study [23] and the issue of being dependent on life-long regular medicine intake while not being physically very sick, might also play a role. As opposed to the patients in stage 3 or 4, who experienced physical improvement, these patients have less clear evidence of the positive side of the medication. Alternatively, one might perceive that there could be a ceiling effect with the WHOQOL-HIVBREF that might occur in the stage 1 and 2 group, with baseline high QOL. However, we were surprised that QOL was not higher among this group at baseline and do not think that there was a significant “ceiling effect” in play during this evaluation.

Meanwhile the intervention improved QOL among participants in the group with severe immunosuppression and opportunistic infections, there were no changes regarding internal AIDS-related stigma scores neither in the intervention nor in the subgroup with different clinical stages. Stigma might be not directly influenced by adherence support measures. In Vietnam there is a strong association between HIV and “social evils” including IDU and sex work as well as fear of HIV transmission [33]. A study carried out in Ho Chi Minh City, Vietnam revealed that PLHIV often faced problems getting a job, perceived unfair treatment in the work place and experienced discrimination from health care providers [34].

The decision to use peer support as an intervention was taken as a result of focus group discussions with patients on ART [23] when ART was newly implemented in Vietnam and the majority of the participants had been or were severely immunosuppressed with opportunistic infections. The effect of peer support on QOL improvement depends on the clinical stages of patients as shown by this study. This randomized controlled trial implemented a common standardized intervention for all patients, independent of clinical staging and severity of disease and, therefore, may have some limitations. However, the findings indicate a need to develop appropriate intervention tools tailored according to the severity of disease at ART initiation to enable contextualization of the support to different strata of the patient population. Based on our results, we cannot recommend a general peer support intervention but rather an intervention targeted to patients with advanced stages of HIV infection. While there seem to have been benefits for the patients in stage 3 and 4, there were no such effects on the patients with less advanced disease. Possibly similar positive effects could be achieved by support to HIV positive clubs of various kinds, encouraging twinning of patients for those who wish, group support meetings at the hospital etc, rather than organized as the individual resource-intensive process presented in this study. With such an approach, patients’ needs could be revaluated on a regular basis. For patients who initiate ART when they are at clinical stage 1 or 2, adherence support via a mobile phone text message may be considered more appropriate than peer support in some settings, as it might be perceived to interfere less with patient privacy. This approach has been applied successfully in several other contexts [35,36] and is now being evaluated in a randomized controlled trial in India [37].

Our results show that the intervention had a positive effect on QOL among those who were at clinical stages 3 and 4. We have not assessed the intervention from a cost effectiveness perspective, but the use of peer-supporters is a comparably low-cost measure, and our recommendation would be for the health system to continue to work with peer-supporters interested in this job, and then to specifically use them for support to patients at clinical stages 3 and 4, who are starting ART.

A number of other independent factors shown to have an impact on QOL in this study have also been demonstrated by other studies. For example, people who experience less stigma are more likely to optimistically assess their QOL in general [17,18,38]. The relationship between co-infection with hepatitis B and/or C and reduced QOL may be due to the fact that the major symptoms of hepatitis B and C are caused by an immune reaction; hence, with improved immunocompetence for patients on ART, hepatitis symptoms may be more pronounced [39-41]. This indicates the need for improved hepatitis management for PLHIV on ART. Patients who have higher education levels will achieve better QOL [10], possibly as they are more integrated in society and may have a better social network of family and friends. It is difficult to explain why those who witnessed the death of a family member due to HIV had better QOL improvement. However, it might have been that they valued their own lives and improved health more after the grim experience of losing a family member to HIV [42].

Contradicting findings from other studies, the findings in this study did not show that reported intravenous drug use, present or prior, influences QOL in any direction [12,43].

This study had some limitations. For example, factors such as employment, income and marital status were only collected at baseline and might not have accurately reflected the influence of these factors on QOL if they changed over time. Other limitations may include a rather high withdrawal rate (17%) and the potential contamination which may have resulted in over or underestimating the effect of the intervention on QOL. However, as the withdrawal rate was similar in the two groups and patients were randomized, these potential effects can be presumed to be similar in the intervention and control groups. By randomizing the 71 communes, to increase the geographical spread, we assumed that the risk for contamination was decreased or at least less common as compared to an individual randomization design. The main objective of the DOTARV project was to assess whether peer support can improve patients’ adherence to ART and decrease treatment failure rates. Adherence could be an influencing factor of QOL in either positive or negative way. Increased adherence could result in greater suppression of the virus and result in increased quality of life [14,15] or greater adherence might be associated with increased adverse effects of medications resulting in decreased quality of life [31]. As this is an ongoing cohort that continues to be followed, it may be possible to assess the co-variation of QOL, adherence and clinical outcomes of the intervention at a later stage. A potential weakness of our study is the fact that a Minimal Clinically Important Difference (MCID) has not been established for the WHOQOL-HIVBREF instruments used. While studies on QOL in relation to HIV and ART are now appearing from different contexts in both low- and middle- income countries, there are clearly contextual differences in indicators, dependent on country of study [44]. Hence, we hope our results will contribute to the further development of this research area.

## Conclusions

The peer support intervention improved QOL after 12 months follow up for patients who were enrolled on ART with severely immunosuppressed condition (clinical stages 3 and 4) but had no impact on QOL improvement for patients enrolled with mild or no clinical symptoms (clinical stages 1 and 2). Neither had the intervention any effect on Internal AIDS-related stigma. To improve QOL for PLHIV on ART, measures to support adherence should be contextualized in accordance with the individual clinical and social needs of the patient.

## **Competing interests**

The authors declare that they have no competing interests.

## **Authors' contributions**

VVT: Study design, data collection, data entry, data analysis, manuscript writing, AP: Study design, research tool developing, manuscript writing, BD: Data entry, manuscript writing, NPH: Study design, data collection, manuscript writing, NTKC: Study design, manuscript writing, HDP: data analysis, manuscript writing, ML: Study design, data analysis, manuscript writing, AT: Study design, data analysis, manuscript writing,GM: Data analysis, manuscript writing. All authors read and approved the final manuscript.

## **Authors' information**

VVT: MD, MPH, PhD student at Division of Global Health, Department of Public Health Sciences, Karolinska Institutet, Stockholm, Sweden; Head of Infectious Disease Department, Uong Bi General Hospital, Vietnam; Researcher in the Health Systems Research Project, Hanoi Medical University, Ha Noi, Vietnam.

AP: MSN, MPH, PhD at Division of Global Health, Department of Public Health Sciences, Karolinska Institutet, Stockholm, Sweden.

ML: MD, PhD, Researcher at Division of Global Health, Department of Public Health Sciences, Karolinska Institutet, Stockholm, Sweden; Senior Researcher of Oxford University Clinical Research Unit, Ha Noi, Vietnam.

AT: MD, PhD, Associate Prof at Division of Global Health, Department of Public Health Sciences, Karolinska Institutet, Stockholm, Sweden.

HDP: PhD, Researcher at Department of Probability and Mathematical Statistics, Institute of Mathematics, Ha Noi, Vietnam.

NPH: MD, PhD, Researcher of Health System Research Project, Ha Noi Medical University, Ha Noi, Vietnam.

NTKC: PhD, Associate Prof, Researcher of Health System Research Project, Ha Noi Medical University, Ha Noi, Vietnam.

GM: PhD, Researcher at Division of Global Health, Department of Public Health Sciences, Karolinska Institutet, Stockholm, Sweden.

BD: MD, Sundsvall-Härnösand Regional hospital, Sundsvall, Sweden.

## Author details

^1^Division of Global Health, Department of Public Health Sciences, Karolinska Institute, Stockholm, Sweden. ^2^Health System Research Project, Ha Noi Medical University, Ha Noi, Vietnam. ^3^Department of Infectious diseases, Uong Bi general hospital, Uong Bi, Quang Ninh, Vietnam. ^4^Department of Probability and Mathematical Statistics, Institute of Mathematics, Ha Noi, Vietnam. ^5^Oxford University Clinical Research Unit, Ha Noi, Vietnam.^6^Sundsvall-Härnösand regional hospital, Sundsvall, Sweden.
